# Relationship between duration of illness and cardiac autonomic nervous activity in anorexia nervosa

**DOI:** 10.1186/s13030-015-0032-6

**Published:** 2015-04-23

**Authors:** Yoshikatsu Nakai, Masatoshi Fujita, Kazuko Nin, Shun’ichi Noma, Satoshi Teramukai

**Affiliations:** Kyoto Institute of Health Sciences, Miyako Bldg. 502, Karasuma Oike Agaru Higashigawa, Nakagyo-ku, Kyoto 604-0845 Japan; Human Health Sciences, Kyoto University Graduate School of Medicine, Kyoto, Japan; Department of Psychiatry, School of Medicine, Kyoto University, Kyoto, Japan; Department of Biostatistics, Kyoto Prefectural University of Medicine Graduate School of Medical Science, Kyoto, Japan

**Keywords:** Anorexia nervosa, Heart rate variability, Low-frequency power, High-frequency power, Cardiac autonomic nervous activity

## Abstract

**Background:**

The mortality rate associated with anorexia nervosa (AN) is high, and death is mainly attributable to cardiac events. A wide range of autonomic nervous system disturbances may be mechanisms underlying the increased cardiovascular mortality and sudden death of patients with AN. Heart rate variability (HRV) has been proven to be a reliable noninvasive method for quantitative assessment of sympathetic and parasympathetic regulation of heart rate (HR). The longer the duration of illness of AN patients, the higher the mortality rate. However, there have been few reports on the relationship between the duration of illness and HRV in AN. Hence, the aims of this study were to compare the cardiac autonomic nervous activity (CANA) of female patients with AN and age-matched female controls and to evaluate the relationship between the duration of illness and the CANA of the AN patients.

**Methods:**

We studied 14 female patients with AN and 22 age-matched healthy women. Beat-to-beat heart rate variability, recorded in a supine position, was investigated using power spectral analysis.

**Result:**

Mean heart rate was positively correlated with normalized high-frequency (HF: 0.15 to 0.40 Hz) power and negatively correlated with the low-frequency (LF: 0.04 to 0.15 Hz)/HF power (LF/HF) ratio of the controls. On the other hand, duration of illness was negatively correlated with normalized HF power and positively correlated with the LF/HF ratio of the AN patients.

**Conclusions:**

These results suggest that, given that the LF/HF ratio is an estimate of cardiac sympathovagal balance, anorectic patients with a long illness duration display lower vagal tone (parasympathetic withdrawal) and high sympathetic tone.

## Background

Although once believed to be a problem primarily limited to female Caucasians, individuals from a variety of racial and ethnic groups are affected by eating disorders [1]. Although “Fushoku-byou”, a form of anorexia nervosa (AN), had been described by physicians of Oriental medicine during the last quarter of the 18th century, modern study of AN in Japan dates from the end of the 1950s. The number of patients with eating disorders in clinical and community samples has increased dramatically in Japan since the 1980s [2,3]. This is an important social health concern in Japan [2], as in Western countries [1].

The mortality rate associated with AN is high (5.9%-18%), and death is mainly attributable to cardiac events in Western countries [4]. When we conducted an outcome study in 233 Japanese women with an eating disorder, followed from 4 to 9 years, 14 (10.2%) of 137 anorectic patients had died before follow-up due to cardiac events (n = 10), pneumonia (n = 1), sepsis (n = 1), multiple organ failure syndrome (n = 1), and suicide (n = 1) [5]. Individuals with AN demonstrate cardiac structural (decreased left ventricular mass and wall thickness), functional (left ventricle dysfunction), and electrical problems (prolonged QT interval and bradycardia) [6]. A wide range of autonomic nervous system (ANS) disturbances may be mechanisms underlying the increased cardiovascular mortality and sudden death of patients with AN [7].

Heart rate variability (HRV) has been proven to be a reliable noninvasive method for quantitative assessment of sympathetic and parasympathetic regulation of heart rate (HR) in different study populations. Mazurak et al. [7] conducted a review of the literature on HRV as a measure of cardiac autonomic function in AN. Twenty papers on HRV in patients with AN were identified and analyzed, revealing three distinct viewpoints regarding changes of ANS functions in patients with AN. The majority of papers identified sympathetic/parasympathetic imbalance with parasympathetic dominance and decreased sympathetic modulation; others could not replicate these findings, but instead described sympathetic dominance; finally, a group of papers could not identify any autonomic functional differences in comparison to control samples.

Our outcome study revealed that the longer the duration of illness of AN patients, the higher the mortality rate [8]. However, there have been few reports on the relationship between the duration of illness and HRV in AN [6,9]. DiVasta et al. [6] reported that anorectic patients with higher heart rates at admission had a longer duration of illness. However, they did not study the HRV of these patients. In a study by Wu et al. [9], there was no significant difference in heart rate between AN patients and controls, but AN patients, who suffered from long term malnutrition, showed a lower sympathetic activity and a higher parasympathetic activity. However, they did not describe the range of the duration of illness of these patients [9].

Hence, the aims of this study were to compare the cardiac autonomic nervous activity (CANA) of female AN patients, with a wide range of duration of illness, and age-matched female controls and to evaluate the relationship between the duration of illness and the CANA of AN patients.

## Methods

### Participants

We studied 14 women referred to Kyoto University Hospital in 2005 who met the DSM-IV criteria [10] for the restricting type of AN. Patients having anxiety disorders and/or emotional disorders were excluded from this study. The duration of illness ranged from 6 to 321 months. Twenty-two age-matched female controls were recruited by public notices at the university center. Interested individuals were screened with a diagnostic checklist by a senior author (YN) to ensure that they did not meet the criteria for a current Axis I disorder and did not report any history of eating disorders or cardiovascular diseases. Ten controls had a low BMI, less than 18.5 kg/m^2^, and twelve had a normal BMI, 18.5 to 25.0 kg/m^2^. There was no significant difference in age, HR or the results of heart rate analyses between the two groups; hence, data of the two groups were combined. Demographic and clinical data are shown in Table 1. All of the subjects gave their written informed consent for the study. Ethical approval for this study was given by the Ethics Committee of Kyoto University Graduate School and Faculty of Medicine.

**Table 1:** **Demographic and clinical data**

	**Anorectic patients (** **n = ** **14)**	**Controls (** **n = ** **22)**	**p value**
	**mean ± ** **SD**	**mean ± ** **SD**	
Age (years)	27.9 ± 9.4	23.2 ± 4.2	0.102
Body mass index (kg/m^2^)	13.2 ± 1.9	18.5 ± 1.7	0.000
Systolic blood pressure (mmHg)	96.1 ± 6.2	103.9 ± 7.3	0.015
Diastolic blood pressure (mmHg)	56.6 ± 5.6	64.3 ± 8.1	0.021
Age of onset (years)	18.4 ± 4.8		
Duration of illness (months)	121.9 ± 105.3		
Serum sodium level (mEq/L)	140.0 ± 1.8		
Serum potassium level (mEq/L)	3.9 ± 0.2		
serum chloride level (mEq/L)	100.0 ± 1.5		

### Procedure

None of the subjects had taken any medications during the month prior to testing. All subjects were asked to refrain from consuming caffeine, alcohol, and tobacco for at least 24 hours prior to testing. Electrocardiogram (ECG) recordings were performed for ten minutes in a supine position after a resting period of 20 min between 10:00 AM and 12:00 AM. Five-minute stationary and artifact-free sections of ECG recordings were selected. The heart rate analyses were carried out using a commercially available computer program (SyneTec, Ela Medical Japan Co., Ltd., Tokyo, Japan). There were no missing values due to artifacts and no form of artifact correction was employed.

The results of spectral analyses were calculated by fast Fourier transformation, according to the user’s manual of SyneTec. Frequency domain measurements included total power (0.04 to 0.40 Hz), low-frequency power (LF: 0.04 to 0.15 Hz), and high-frequency power (HF: 0.15 to 0.40 Hz). Following the recommendations by the Task Force on HRV Research [11], normalized HF power (=100 × HF power/total power), normalized LF power (=100 × LF power/total power), and LF/HF ratio were calculated. We used normalized HF power as an index of cardiac parasympathetic activity and LF/HF ratio as an index of cardiac sympathovagal balance. Higher values of LF/HF reflect sympathetic dominance or parasympathetic withdrawal, while lower ones reflect sympathetic withdrawal or parasympathetic dominance.

### Statistical analysis

All results are expressed as the mean ± SD. The statistical analysis was performed with SPSS 13.0. Statistical comparisons between AN patients and controls were performed by Student’s unpaired *t* test. Spearman’s correlations were used to evaluate the relationship among different variables. P < 0.05 was considered statistically significant.

## Results

There was no significant difference in age between the AN patients and controls. However, there were significant differences in body mass index (BMI), systolic blood pressure, diastolic blood pressure, and pulse rate, as shown in Table 1.

There was a significant difference in the mean heart rate (HR) between AN patients and controls; however, there was no significant difference in normalized LF power, normalized HF power, or LF/HF ratio, as shown in Table 2.

**Table 2:** **Mean heart rate and frequency domain measurements of heart rate variability**

	**Anorectic patients (** **n = ** **14)**	**Controls (** **n = ** **22)**	**p value**
	**mean ± ** **SD**	**mean ± ** **SD**	
Heart rate	56.1 ± 12.5	70.0 ± 15.1	0.007
Normalized low-frequency power (nu)	38.8 ± 17.2	34.7 ± 12.5	0.422
Normalized high-frequency power (nu)	42.3 ± 16.3	38.8 ± 20.1	0.395
Low-/high-frequency power ratio	1.2 ± 1.1	1.3 ± 1.1	0.788

The mean heart rate (HR) of the controls was negatively correlated with normalized HF power and positively correlated with LF/HF ratio (Table 3). On the other hand, duration of illness of the AN patients was negatively correlated with normalized HF power and positively correlated with normalized LF power and LF/HF ratio (Table 4). No outliers were found on visual inspection of the scatter plots of significant correlations, with the exception of one in a scatter plot for the duration of illness and LF/HF ratio of the AN patients. When this outlier was removed, the *r* value changed from 0.784 to 0.763.

**Table 3:** **Correlations among various variables:** **controls**

	**Mean HR**	**LFn**	**HFn**	**LF**/**HF**	**BMI**
Mean HR		-.008	-.616**	.554**	-.397
LFn			-.207	.526*	.164
HFn				-.905**	.272
LF/HF					-.187

**: p < 0.01, *: p < 0.05.

HR: heart rate.

LFn: normalized low-frequency power.

HFn: normalized high-frequency power.

LF/HF: low-/high-frequency power ratio.

BMI: body mass index.

**Table 4:** **Correlations among various variables:** **patients with anorexia nervosa**

	**Mean HR**	**LFn**	**HFn**	**LF**/**HF**	**BMI**	**Duration of illness**
Mean HR		-.004	-.427	.176	.172	.109
LFn			-.600*	.895**	-.156	.725**
HFn				-.824**	.020	-.667**
LF/HF					-.134	.784**
BMI						.075

**: p < 0.01, *: p < 0.05.

HR: heart rate.

LFn: normalized low-frequency power.

HFn: normalized high-frequency power.

LF/HF: low-/high-frequency power ratio.

BMI: body mass index.

## Discussion

In the present study, mean HR of the controls was negatively correlated with normalized HF power, an index of cardiac parasympathetic activity, and positively correlated with LF/HF ratio, an index of cardiac sympathovagal balance. These results suggest that the mean HR was regulated appropriately by the ANS function of the controls [11].

On the other hand, the duration of illness of the AN patients was negatively correlated with normalized HF power and positively correlated with LF/HF ratio. This indicates that parasympathetic tone was stronger in AN patients with a short illness duration. The higher parasympathetic tone of AN patients with a short illness duration could be considered to be an adaptive response to caloric deprivation, as previously described [7,9,12]. On the other hand, given that the LF/HF ratio is an estimate of cardiac sympathovagal balance, AN patients with a long illness duration displayed lower vagal tone (parasympathetic withdrawal) and high sympathetic tone. The sympathetic/parasympathetic imbalance with sympathetic predominance of AN patients with a long illness duration could be detrimental and contribute to the higher cardiovascular mortality risk of these patients. In fact, increased sympathetic tone has been shown to cause an increased risk of sudden cardiac death by cardiac failure [13]. The findings of the present study differ from those of a previous study. Wu et al. [9] reported that the duration of illness of AN patients (n = 14) had a significant negative relationship to normalized LF power, but it had a significant positive relationship to normalized HF power. There was no significant relationship between the duration of illness and the LF/HF ratio of these patients. Since Wu et al. did not describe the range of duration of illness, a possible explanation for the discrepancy in the results between our study and their study may be a difference in the range of duration of illness.

A limitation of the current study was that our sample is small and thus limits our ability to detect small effect sizes for comparisons, owing to the limited statistical power. This study was cross-sectional rather than longitudinal, so the findings of the duration of illness for HRV should be interpreted with caution. We should also have measured the respiration rate to estimate HF power more precisely.

In summary, the findings of the present study suggest that, given that the LF/HF ratio is an estimate of cardiac sympathovagal balance, anorectic patients with a long illness duration displayed lower vagal tone (parasympathetic withdrawal) and high sympathetic tone.
